# Novel Potassium-Competitive Acid Blocker, Tegoprazan, Protects Against Colitis by Improving Gut Barrier Function

**DOI:** 10.3389/fimmu.2022.870817

**Published:** 2022-05-25

**Authors:** Mijeong Son, I Seul Park, Soochan Kim, Hyun Woo Ma, Ji Hyung Kim, Tae Il Kim, Won Ho Kim, Jaeyong Han, Seung Won Kim, Jae Hee Cheon

**Affiliations:** ^1^Department of Internal Medicine and Institute of Gastroenterology, Graduate School of Medical Science, Brain Korea 21 Project, Yonsei University College of Medicine, Seoul, South Korea; ^2^Department of Internal Medicine, Cha Ilsan Medical Center, CHA University, Goyang, South Korea; ^3^Severance Biomedical Science Institute, Yonsei University College of Medicine, Seoul, South Korea

**Keywords:** *Bacteroides vulgatus*, inflammatory bowel disease, potassium-competitive acid blocker, proton pump inhibitor, gut barrier function

## Abstract

Inflammatory bowel disease (IBD) is a chronic immune-mediated disorder characterized by prolonged inflammation of the gastrointestinal tract. IBD can result from gut barrier dysfunction, altered gut microbiota, and abnormal intestinal immunity induced by environmental factors in genetically susceptible individuals. Proton pump inhibitors (PPIs) such as rabeprazole are frequently employed for gastric acid inhibition. However, long-term PPI administration can alter the intestinal microbiome composition, possibly worsening IBD severity. The present study revealed that tegoprazan, a potassium-competitive acid blocker, significantly improved colitis in mice and enhanced the intestinal epithelial barrier function. Tegoprazan alleviated gut microbiota dysbiosis and enhanced the growth of *Bacteroides vulgatus*. In turn, *B. vulgatus* alleviated intestinal inflammation by inhibiting epithelial adhesion of pathogenic bacteria. Unlike rabeprazole, tegoprazan did not induce gut dysbiosis. Our findings provide novel insights into the potential role of tegoprazan as an intestinal protectant for IBD and as a therapeutic agent for gastric acid-related diseases.

## Introduction

Inflammatory bowel diseases (IBD), including ulcerative colitis (UC) and Crohn’s disease (CD), are immune-mediated diseases characterized by progressive inflammation affecting the gastrointestinal tract (1). Reportedly, the incidence and prevalence of IBD are growing globally, across Asia, North America, and Europe; however, the etiology of IBD remains unclear, most frequently attributed to abnormal immune responses and barrier dysfunction in the gut, catalyzed by the genetic susceptibility of the host (2, 3).

The gastrointestinal tract harbors a complex and large population of microorganisms. This microbiota plays a fundamental role in maintaining immunity, homeostasis, and protection against pathogens. The microbiota affords several beneficial effects to the host, including metabolism, nutrient synthesis, and immune system development. Furthermore, it helps maintain the intestinal barrier integrity by enhancing the production of mucus, antimicrobial peptides, and junction proteins (3). However, an imbalance in the gut microbiota, also known as “dysbiosis,” can lead to epithelial dysfunction, abnormal intestinal permeability, and intestinal inflammation. In line with these findings, epithelial defects that prolong chronic mucosal inflammation in IBD have been well documented (3).

Proton pump inhibitors (PPIs) are used worldwide to treat acid-related disorders, such as gastroesophageal reflux disease (GERD) and *Helicobacter pylori* infection. PPIs inhibit H^+^, K^+^-adenosine triphosphatase (H^+^/K^+^-ATPase) irreversibly in the parietal cells (4). Although PPIs possess a good safety profile, long-term PPI use has been associated with adverse effects, including bone fractures (5), pneumonia (6), *Clostridium difficile* infection (7), and imbalanced gut microbiota composition (8). Furthermore, recent studies have indicated that long-term PPI usage reportedly exacerbates the disease severity in patients with IBD and increases the risk of IBD-related hospitalization or surgery (9). The precise cause of these adverse effects remains unknown, but the reduced secretion of gastric acid has been deemed a significant risk factor (10). PPIs reduce stomach acidity, allowing the survival of microbes typically killed by the gastric environment, which can increase the risk of gut dysbiosis.

To overcome limitations associated with PPI therapy, including the duration of effect, irreversibility, and side-effects, potassium-competitive acid blockers (P-CABs), a new class of acid suppressants, have been developed (11). Unlike PPIs, P-CABs do not require the acid activation process to inhibit gastric H^+^/K^+^-ATPase reversibly (12). In addition, P-CABs demonstrate a more potent and longer-lasting effect on gastric acid suppression than traditional PPIs such as rabeprazole and omeprazole (13). Moreover, recent studies reported that regular use of PPIs was associated with an increased risk of IBD and its subtypes (14) and has microbiome-related side effects in humans and mice (15, 16). Tegoprazan, a recently launched P-CAB, is widely prescribed to treat GERD and peptic ulcers (17). Although it has a stronger acid inhibiting effect, its different mechanisms of action might differently affect the intestinal microbiome or epithelial tight junction compared with traditional PPIs such as rabeprazole. Potassium channels are involved in various cellular functions and cell to cell communications. However, whether P-CAB affects the severity of IBD or dysbiosis needs to be comprehensively investigated.

Herein, we aimed to clarify whether tegoprazan could be a potential intestinal protectant against IBD *via* tight junction and microbiome modulations and investigated the therapeutic effects of tegoprazan against colitis in a mouse model. Furthermore, we investigated the mechanism through which tegoprazan ameliorates colitis and modulates the microbiome, as well as whether PPI-induced worsening of colon damage and dysbiosis can be attributed to the acid-suppressive effect or the microbiome.

## Materials and Methods

### Animals

Male C57BL/6 mice (6−8 weeks old) were purchased from OrientBio (Sungnam-si, Gyeonggido, South Korea) and acclimatized for 1 week before starting the experiment. Mice were maintained at an ambient temperature of 22°C on a 12-h light/dark cycle in a specific pathogen-free facility.

All experiments using animals were reviewed and approved by the Institutional Animal Care and Use Committee of Yonsei University (Approval No. 2018-0304, 2020-0160), and all methods were performed according to the guidelines and regulations of the IACUC.

After sacrificing animals on Day 5 in the DNBS model and Day 9 in the DSS model, blood was collected from the hearts of anesthetized mice, and the entire colon and the cecal contents from the cecum were harvested for further investigations, snap-freezed, and stored in -70°C before use. The cecal tissues were washed with ice-cold phosphate-buffered saline (PBS) three times and cut using scissors 5 mm in size before snap-freezing.

### Histological Analysis

Colon tissues were fixed in 10% neutral formalin solution overnight, embedded in paraffin, and stained with periodic acid-Schiff reagent (PAS). Images were acquired using a light microscope (Olympus BX41; Olympus Optical, Tokyo, Japan). The severity of symptoms was determined by scoring the extent of bowel wall thickening, crypt damage, and inflammatory cell infiltration (summarized in **Supplementary Table S2**) (18). Goblet cell loss in colon tissues was evaluated using ImageJ software (ver. 1.53e; National Institutes of Health, Bethesda, MD).

Quantitative reverse-transcription polymerase chain reaction (qRT-PCR), immunohistochemistry (IHC), metagenomic analysis, immunofluorescence analysis, western blotting, and flow cytometric analysis are described in the Supporting Material.

### *In Vivo* Intestinal Permeability Assay

Intestinal permeability was evaluated by measuring paracellular permeability to 4 kDa fluorescein isothiocyanate (FITC)-dextran (Sigma-Aldrich) on the day of sacrifice. Mice were orally administered 150 μL of 80 mg/mL FITC-dextran, and blood was collected 4 h after administration. Fluorescence intensity was measured using a fluorescent microplate reader (excitation: 490 nm, FITC emission: 520 nm; Varioskan Flash; Thermo Fisher Scientific).

### Cell Culture and Treatment

Human colon carcinoma cell lines, HT-29 (HTB-38™, Korea Cell Line Bank, Seoul, South Korea) and Caco-2 [HTB-37™, American Type Culture Collection (ATCC), Manassas, VA], were maintained at 37°C in RPMI 1640 (SH30027.FS, HyClone^™^, Logan, UT) containing 10% heat-inactivated fetal bovine serum (FBS) (26140-079, Ab Frontier, Seoul, Korea) and 1% penicillin-streptomycin solution (CA005-100, GenDEPOT, Katy, TX) in a humidified incubator with 5% CO_2_. Caco-2 cells were cultured in Dulbecco’s modified Eagle’s medium (SH30243.FS, HyClone^™^) containing 10% FBS and 1% penicillin-streptomycin solution. Cell viability was assessed by trypan blue staining under a microscope. Caco-2 cells were used to assess intestinal barrier function, with barrier damage induced by treatment with 40 ng/mL tumor necrosis factor (TNF)-α (210-TA, R&D Systems, Minneapolis, MN) for 48 h.

All samples were harvested at 4 h for qRT-PCR analysis, at 24 h for Western blotting, and at 48 h for immunostaining after treatment.

### *In Vitro* Intestinal Permeability Assay

Next, to evaluate the intestinal epithelial barrier function, Caco-2 cells were plated into the upper chamber of the Transwell system (0.4 μm pore, 3460, Corning, NY). Epithelial permeability was assessed by the analysis of transepithelial electrical resistance (TEER) and the paracellular flux of FITC-dextran (FD4, Sigma-Aldrich). The electrical resistance of the Caco-2 cell monolayers cultured in the Transwell chamber was assessed using a Millicell-ERS instrument (Millipore, Bedford, MA). FITC-dextran was added to the upper chamber at a final concentration of 1 mg/ml. Two hours after the addition of FITC-dextran, the medium from the lower chamber was collected, and fluorescence intensity was measured using a fluorescence microplate reader (Varioskan Flash 3001, Thermo Fisher Scientific).

### Flow Cytometry and Cytometric Bead Array (CBA) Analysis

Plasma cytokine concentrations (Il-6 and Tnf-α) were measured using the CBA Mouse Th1/Th2/Th17 Cytokine Kit (560485, BD Biosciences, San Jose, CA), according to the manufacturer’s protocol. Samples were analyzed by flow cytometry (FACS Verse, BD Biosciences). Cytokine levels were normalized to the total protein concentration.

For flow cytometric analysis, single-cell suspensions (1×10^6^ cells) were blocked with 2.5% normal mouse and rat serum in FACS buffer (DPBS containing 0.1% BSA) and stained for 30 min at 4°C with the appropriate antibodies. The antibodies used included monoclonal anti-CD25-PerCP-Cyanine 5.5 (PC61.5, 560503), anti-Foxp3-PE (150D/E4, 12-4774-42), and anti-CD4-FITC (GK1.5, 11-0041-82) antibodies purchased from eBioscience (San Diego, CA), and monoclonal anti-CD3-V500 (500A2, 560771) antibodies purchased from BD Biosciences. For intracellular staining, a Foxp3/Transcription factor staining buffer set was purchased from eBioscience. Data were acquired using a FACSVerse flow cytometer (BD Biosciences) and analyzed using FlowJo software (Tree Star, San Carlos, CA).

### Bacterial Strains and Growth Conditions

*Bacteroides vulgatus* strains were kindly provided by Dr. Sangsun Yoon from Yonsei University. *Salmonella enterica subsp. enterica serovar Typhimurium* (*S. typhimurium*) GFP strains were purchased from ATCC (ATCC 14028GFP). *Bacteroides vulgatus* strains were cultivated in Gifu anaerobic medium (GAM) broth (MB-G0826, KisanBio, Seoul, Korea) under anaerobic conditions. *Salmonella typhimurium* strains were routinely grown at 37°C in nutrient broth containing 100 µg/ml ampicillin under aerobic conditions.

Bacterial adhesion assay is described in the Supporting Material.

### Statistical Analysis

GraphPad Prism 5.0 software (GraphPad Inc., La Jolla, CA) was used for statistical analyses. We tested normality of data and the significance of the differences between the test conditions was assessed using two-way or one-way analysis of variance (ANOVA) followed by Bonferroni *post hoc* test or Dunnett’s multiple comparison test for multiple comparisons. Statistical significance was set at *p* < 0.05.

## Results

### Tegoprazan Alleviates the Severity of Dinitrobenzene Sulfonic Acid (DNBS)-Induced Colitis

**Figure 1:**
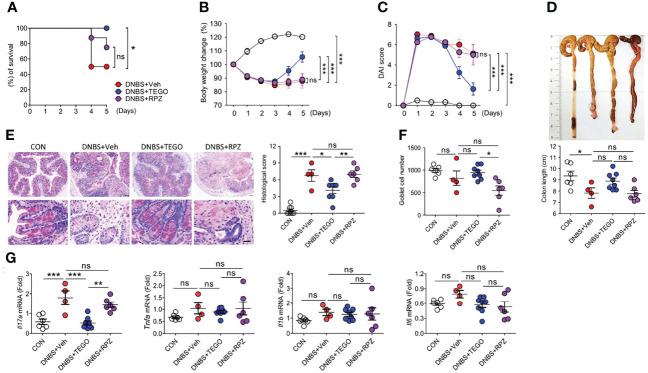
Tegoprazan alleviates DNBS-induced colitis. Colitis was induced in 8-week-old male mice by rectally administering DNBS (5 mg in 100 µL of 50% ethanol). The mice were allocated to four groups: CON (n = 6), DNBS+Veh, DNBS+TEGO, and DNBS+RPZ (each n = 8). Tegoprazan (30 mg/kg) and rabeprazole (30 mg/kg) were administered orally twice daily throughout the experiment. All drugs were dissolved in 0.5% (w/v) methylcellulose. After sacrificing animals on Day 5, the entire colon from anesthetized mice was harvested for further investigations. The detailed methods are described in the Materials and Methods section. **(A)** Survival rate from survival model. Survival analyses were performed using Kaplan-Meier plots for overall survival and differences were compared using a log-rank test. **(B)** Body weight change. **(C)** Disease activity index (DAI). **(D)** Representative images of colons and analysis of colon length. **(E)** Representative images following periodic acid-Schiff (PAS)-staining and histological score. Scale bars = 20 μm. **(F)** Goblet cell number. **(G)** Gene expression levels of proinflammatory cytokines (*Il17*, *Tnfa*, *Il1b*, and *Il6*). Data represent the mean of triplicate real-time quantitative RT-PCR. For **(D–G)**, CON (n = 6), DNBS+Veh (n = 4), DNBS+TEGO, and DNBS+RPZ (each n = 6). Data represent the mean ± standard error of the mean (S.E.M). Significance is indicated by *p < 0.05, **p < 0.01, and ***p < 0.001 using two-way ANOVA followed by Bonferroni *post hoc* test **(B, C)** or one-way ANOVA followed by Dunnett’s multiple comparison test **(D–G)**. ns, not significant; CON, treated with ethanol; DNBS, dinitrobenzene sulfonic acid; Veh, treated with vehicle; TEGO, treated with tegoprazan; RPZ, treated with rabeprazole.

DNBS- and DSS-induced colitis models have been widely used as mouse models of colitis. DNBS-induced colitis presents a phenotype similar to that of human CD (19). Rectal administration of DNBS in the vehicle-treated (DNBS+Veh group) or rabeprazole (DNBS+RPZ group) groups rapidly triggered severe diarrhea, decreased mobility, and induced weight loss, resulting in significant mortality (**Figures 1A–C**). More than 50% of the mice died in the DNBS+Veh group, whereas 100% survival was noted in tegoprazan-treated mice (DNBS+TEGO group) (**Figure 1A**, *P* = 0.048 vs. DNBS+Veh). Although the DNBS+TEGO group exhibited similar weight loss and disease activity index (DAI) scores until Day 3, these symptoms were significantly relieved post-Day 3 when compared with the DNBS+Veh or DNBS+RPZ groups (**Figures 1B, C****)**. Additionally, the DNBS-induced reduced colon length was alleviated by tegoprazan treatment (**Figure 1D**).

The colon of the DNBS+Veh group exhibited significant crypt transformation, mucosal immune cell infiltration, and perforation, as revealed by histological analysis. In contrast, tegoprazan treatment significantly reduced colonic damage (**Figures 1E, F****)**. Proinflammatory cytokines play an essential role in the pathogenesis of IBD (20). qRT-PCR was performed to determine the impact of tegoprazan on inflammatory cytokines. The DNBS+TEGO group demonstrated reduced mRNA expression levels of proinflammatory cytokines, especially interleukin-17 (*Il17*), when compared with the DNBS+Veh group (**Figure 1G**). IL-17 receptor (IL-17R) signaling plays a significant role in the development of TNBS-induced colitis (21). However, in the DNBS+RPZ group, rabeprazole failed to afford protection against DNBS-induced colitis, with significantly reduced goblet cell numbers detected when compared with the DNBS+TEGO group. Collectively, these results confirmed that tegoprazan affords protection against DNBS-induced colon inflammation; this protection was not observed with rabeprazole.

### Tegoprazan Attenuates DSS-Induced Colitis and Proinflammatory Responses

**Figure 2:**
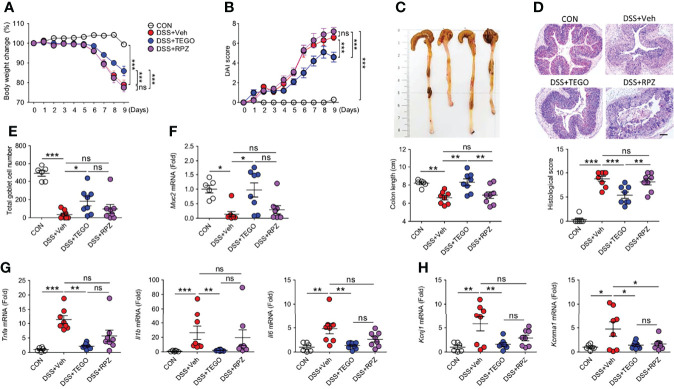
Tegoprazan ameliorates colon damage in DSS-induced colitis. Colitis was induced in 8-week-old male mice by administering 2% DSS in distilled drinking water for 5 days, followed by normal drinking water for 4 days. Tegoprazan (30 mg/kg) and rabeprazole (30 mg/kg) were administered orally twice daily throughout the experiment. All drugs were dissolved in 0.5% (w/v) methylcellulose. After sacrificing animals on Day 9, the entire colon from anesthetized mice was harvested for further investigations. The detailed methods are described in the Materials and Methods section. **(A)** Body weight change. **(B)** Disease activity index (DAI). **(C)** Colon lengths and representative images. **(D)** Representative image following periodic acid-Schiff (PAS)-staining and histological score. Scale bars = 200 μm. **(E)** Total goblet cell number. **(F)** Gene expression level of mucin (*Muc2*). **(G)** Gene expression level of proinflammatory cytokines (*Tnfa*, *Il1b*, and *Il6*). **(H)** Gene expression level of secretory K^+^ channel genes (*Kcnj1* and *Kcnma1*). Data represent the mean of triplicate real-time quantitative RT-PCR. Data represent the mean ± standard error of the mean (S.E.M): For **(A–E)** CON (n = 6), DSS+Veh, DSS+TEGO, and DSS+RPZ (each n = 8). For **(F, G)** CON (n = 6), DNBS+Veh (n = 4), DNBS+TEGO (n = 6), and DNBS+RPZ (n = 6). Significance is indicated by *p < 0.05, **p < 0.01, and ***p < 0.001 using two-way ANOVA followed by Bonferroni *post hoc* test **(A, B)** or one-way ANOVA followed by Dunnett’s multiple comparison test **(C–H)**. ns, not significant; CON, normal control; DSS, treated with dextran sulfate sodium; Veh, treated with vehicle; TEGO, treated with tegoprazan; RPZ, treated with rabeprazole.

DSS-induced colitis presents a phenotype similar to that of human UC. To compare the anti-colitic effect of tegoprazan with that of filgotinib, a Janus kinase inhibitor, colitis was induced by administering 2.5% DSS. Notably, 2 of the 13 mice in the vehicle and filgotinib-treated groups (DSS+FILGO) died during the experiment, whereas all mice in the tegoprazan group survived (**Supplementary Figure S1A**). Herein, filgotinib and tegoprazan significantly restored the body weight of DSS-treated mice (**Supplementary Figure S1B**). Tegoprazan administration significantly improved disease manifestations in terms of DAI, colon length, histopathology, and goblet cell score (**Supplementary Figures S1C, F**), as well as inflammation-related markers (*Muc2*, *Il1b*, and *Il6*) in colon tissues (**Supplementary Figures S1G, H**) when compared with the DSS+Veh group. To further examine the anti-inflammatory effects of tegoprazan and rabeprazole, a milder mouse colitis model was induced by administering 2% DSS in distilled drinking water for 5 days. Compared with the control group, DSS treatment resulted in colitis symptoms such as weight loss, diarrhea, bloody stools, and colon shortening (**Figures 2A–C**). However, these symptoms were alleviated in tegoprazan-administered mice. The relative body weight was significantly higher in the DSS+TEGO group than in the DSS+Veh group from Day 6 of the recovery phase (**Figure 2A**). Furthermore, the elevated DAI score in the DSS+Veh group was markedly reduced in the DSS+TEGO group (**Figure 2B**). The colon length of the DSS+Veh group (6.6 ± 0.2 cm) was significantly reduced when compared with that of the control group (8.2 ± 0.2 cm); tegoprazan alleviated this phenomenon (8.3 ± 0.4 cm) (**Figure 2C**). However, no significant differences were observed between the DSS+Veh and the rabeprazole-treated groups in terms of weight loss, DAI, and colon length.

Tegoprazan-treated mice exhibited markedly ameliorated intestinal injury, reduced inflammatory cell infiltration, and significantly decreased histological scores when compared with the DSS+Veh group. However, the DSS+RPZ group displayed no marked protection against colon damage, as indicated by the high histological scores (**Figure 2D**). In addition, the DSS+Veh group exhibited severe goblet cell loss, whereas the DSS+TEGO group displayed significantly increased goblet cell numbers (**Figure 2E**). In line with the goblet cell analysis, the mRNA expression level of *Muc2* in the colon was significantly upregulated in the tegoprazan-treated group when compared with the DSS+Veh group (**Figure 2F**).

The mRNA expression levels of *Tnfa*, *Il1b*, and *Il6* were significantly increased in the DSS+Veh group. Conversely, the levels of these cytokines were significantly reduced in the tegoprazan-treated group, similar to those observed in the control group (**Figure 2G**). In addition, the mRNA expression levels of *Kcnj1* and *Kcnma1*, secretory K^+^ channel genes, were upregulated in the DSS+Veh group when compared with the control group; however, tegoprazan or rabeprazole administration reduced these DSS-induced increased expression levels (**Figure 2H**), indicating that tegoprazan relieves colitis by suppressing the expression of proinflammatory factors, but not K^+^ channels. Collectively, these findings suggest that tegoprazan ameliorates DSS-induced colitis, although rabeprazole does not exhibit this effect.

### Tegoprazan Prevents Intestinal Permeability and Loss of Tight Junction Proteins

**Figure 3:**
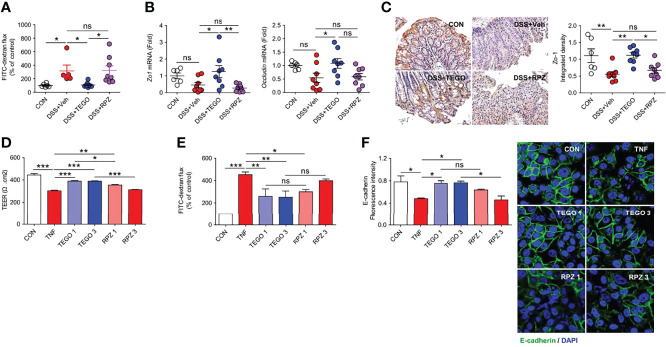
Tegoprazan restores intestinal epithelial barrier function. **(A–C)** *In vivo* intestinal permeability assay using the DSS model was described in online methods: CON (n = 6), DSS+Veh, DSS+TEGO, and DSS+RPZ (each n = 8). **(A)** FITC-dextran flux (arbitrary unit), plotted as percentage of control. **(B)** Gene expression level of *Zo1* and *Occludin*. Data represent the mean of triplicate real-time quantitative RT-PCR. **(C)** Representative immunohistochemical images and densitometric analysis of Zo-1 protein. **(D–F)** Caco-2 cell monolayers in a Transwell plate or culture plate were treated with TNF-α (40 ng/mL) for 48 h to disrupt the epithelial barrier. **(D)** TEER measurement. **(E)** FITC-dextran flux (arbitrary unit), plotted as a percentage of control. **(F)** Quantification of the fluorescence intensity of E-cadherin (arbitrary unit) and representative image of E-cadherin. Data represent the mean ± standard error of the mean (S.E.M) of 3 independent experiments. Significance is indicated by *p < 0.05, **p < 0.01, and ***p < 0.001 using one-way ANOVA followed by Dunnett’s multiple comparison test **(A−D)** or two-way ANOVA followed by Bonferroni *post hoc* test **(E)**. ns, not significant; CON, treated with DMSO; TNF-α, tumor necrosis factor-α; TEGO 1, treated with TNF-α and 1.0 μM tegoprazan; TEGO 3, treated with TNF-α and 3.0 μM tegoprazan; RPZ 1, treated with TNF-α and 1.0 μM rabeprazole; RPZ 3, treated with TNF-α and 3.0 μM rabeprazole; Tregs, regulatory T cells; TEER, transepithelial electrical resistance; FITC, fluorescein isothiocyanate.

The impaired epithelial barrier facilitates increased intestinal permeability, leading to the development of chronic inflammation (22). Because we obtained more significant effects of tegoprazan in the DSS-induced colitis model, we focused on the gut barrier function in the DSS-induced colitis model and investigated microbiome modulation by tegoprazan for a longer duration of days, resulting in immune responses altered by mucosal barrier function in the colonic epithelium. To characterize the protective effects of tegoprazan on epithelial permeability, intestinal permeability was measured using the fluorescein isothiocyanate (FITC)-dextran assay in the DSS mouse model, in which disruption of the intestinal epithelial barrier is induced by DSS. As shown in **Figure 3A**, intestinal permeability was markedly increased in the DSS+Veh and DSS+RPZ groups when compared with the DSS+TEGO group. As tight junctions regulate intestinal epithelial permeability, mRNA levels of tight junction-specific molecules, including *Zo1* and *Occludin*, were analyzed. qRT-PCR results revealed that mRNA levels of *Zo1* and *Occludin* were significantly higher in the DSS+TEGO group than those in the DSS+Veh group (**Figure 3B**). Furthermore, the protein levels of Zo-1 were investigated using immunohistochemistry. The DSS+Veh group exhibited significantly lower Zo-1 protein levels than the control group. Consistent with the mRNA level, the protein level of Zo-1 was remarkably ameliorated in the tegoprazan-treated group (**Figure 3C**). These findings suggest that tegoprazan protects the intestinal epithelial tight junction barrier and inhibits the increase in intestinal permeability.

To further confirm molecular mechanisms underlying the protective role of tegoprazan in intestinal barrier function, we employed an *in vitro* Caco-2 cell culture system (23), widely employed to assess intestinal epithelial barrier function. Accordingly, Caco-2 cell monolayers were treated with TNF-α (40 ng/mL) for 48 h to disrupt the epithelial barrier, followed by the measurement of the TEER. Tegoprazan administration significantly suppressed the TNF-α-induced reduction in TEER levels; however, rabeprazole failed to demonstrate a similar effect (**Figure 3D**). In addition, the paracellular permeability of Caco-2 cell monolayers in Transwell was evaluated using the FITC-dextran assay. TNF-α significantly increased the FITC-dextran flux, which significantly decreased following tegoprazan treatment (**Figure 3E**). Consistently, immunostaining with anti-E-cadherin or ZO-1 antibodies revealed that tegoprazan markedly increased the adhesion and tight junction protein production in the Caco-2 monolayer; conversely, rabeprazole displayed a decreasing trend in these proteins (**Figure 3F**). Furthermore, TNF-α treatment decreased culture media pH, whereas both tegoprazan and rabeprazole restored pH levels to the level of the vehicle-treated control; this indicated that change in pH induced by tegoprazan or rabeprazole did not impact the epithelial barrier function of Caco-2 cell monolayers (**Supplementary Figure S2**). These data indicate that tegoprazan suppresses TNF-α-induced disruption of epithelial barrier function, whereas rabeprazole does not mediate this action. These *in vitro* results are consistent with the *in vivo* observation that tegoprazan reduces DSS-induced colitis by maintaining high junction integrity of the epithelial mucosa.

### Tegoprazan Enriches *Bacteroides Vulgatus* Without Influencing Pathogenic Bacteria

**Figure 4:**
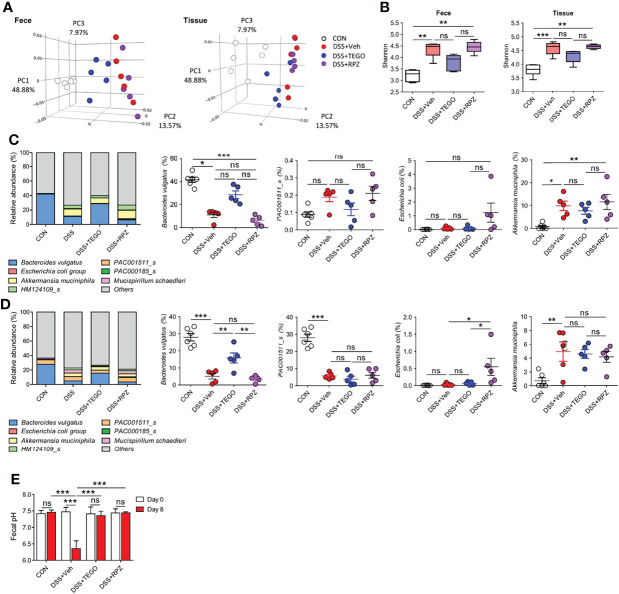
Tegoprazan alters microbiota and prevents dysbiosis in colitis. Gut microbiome composition in feces and tissue was investigated using 16S rRNA sequencing: CON (n = 6), DSS+Veh, DSS+TEGO, and DSS+RPZ (each n = 5). **(A–C)** Effect of tegoprazan on beta **(A)** and alpha diversity **(B)** indices in DSS-induced colitis. **(A)** Principal coordinate analysis (PCoA). **(B)** Shannon index. Box plots with median, 25th/75th percentiles and whiskers from minimum to maximum, and individual values plotted. **(C, D)** Microbial community bar plot by species in fecal **(C)** and tissue **(D)** samples and the abundance ratio of *Bacteroides vulgatus*, *PAC001511_s*, *Escherichia coli*, and *Akkermansia muciniphila*. **(E)** Fecal pH: DSS+RPZ (n = 5), CON, DSS+Veh, and DSS+TEGO (each n = 4). Data represent the mean or mean ± standard error of the mean (S.E.M). Significance is indicated by *p < 0.05, **p < 0.01, and ***p < 0.001 using one-way ANOVA followed by Dunnett’s multiple comparison test. ns, not significant; CON, normal control; DSS, treated with dextran sulfate sodium; Veh, treated with vehicle; TEGO, treated with tegoprazan; RPZ, treated with rabeprazole.

To determine whether tegoprazan attenuates colitis by altering the gut microbiota, a 16S rRNA gene analysis of cecal contents and tissue was undertaken. Principal coordinate analysis revealed that the microbiota structure of the tegoprazan-treated group differed significantly from that of the DSS+Veh group in both fecal and tissue samples, whereas the DSS+RPZ and DSS+Veh groups presented markedly similar microbiota structures (**Figure 4A**). The DSS+TEGO group showed significantly increased microbiota diversity and enrichment in fecal and cecal tissue samples on assessing the Shannon and Simpson indices, with a decreasing trend observed in the cecal tissue samples of the DSS+RPZ group (**Figure 4B**; **Supplementary Figure S3A**). Interestingly, the DSS+Veh and DSS+RPZ groups revealed decreased microbiota diversity, with elevated levels of Firmicutes in fecal and cecal tissues when compared with the CON or DSS+TEGO group (**Figure 4B**; **Supplementary Figure S3**). These results may be attributed to the vehicle effects, elevating Firmicutes under colitic conditions. Indeed, beneficial bacteria such as *Lactobacillus* spp. (24), belonging to the phylum Firmicutes, remained unelevated in the DSS+Veh and DSS+RPZ groups when compared with the DSS+TEGO group (**Supplementary Figure S4**). Nevertheless, tegoprazan reverted the microbiome to the normal state.

The normal gut microbiota consists of two major phyla, namely Firmicutes and Bacteroidetes (25). At the phylum level, the relative abundance of Bacteroidetes decreased in the cecal contents and tissues of DSS and DSS+RPZ groups (**Supplementary Figures S3D, E**). However, tegoprazan administration restored the DSS-reduced abundance ratio of Bacteroidetes in cecal contents and tissues, similar to that observed in the control group samples. Furthermore, at the species level, the bacterial composition of the DSS+TEGO group differed from that of the DSS+Veh and DSS+RPZ groups (**Figures 4C, D****)**. Notably, while *B. vulgatus* was the most plentiful species in control mice (42% in feces and 28% in tissues), DSS decreased the abundance ratio of *B. vulgatus* in the intestine (11% in feces and 5% in tissues), and tegoprazan administration explicitly increased the abundance ratio of *B. vulgatus* in both cecal contents and tissues (29% and 16%, respectively). However, rabeprazole did not impact the abundance of *B. vulgatus*. In contrast, rabeprazole significantly increased the relative abundance of the phylum Proteobacteria, especially of *Escherichia coli* (**Figures 4C, D**; **Supplementary Figures S3, S4**). Additionally, consistent with the *in vitro* data presented in **Supplementary Figure S2**, the DSS+Veh group demonstrated a significantly lower fecal pH at the end of the experiment when compared with the starting point (**Figure 4E****)**. In contrast, the fecal pH in the DSS+TEGO group or the DSS+RPZ group was unaltered, indicating that pH does not influence the effect of tegoprazan or rabeprazole. Collectively, these results indicated that tegoprazan promotes the proliferation of certain bacteria such as *B. vulgatus* independent of pH changes and is not associated with the growth of the pathogenic bacteria such as Proteobacteria which can be induced by an acid-suppressive effect or the specific drug-class effect of PPIs (8, 26).

### *Bacteroides Vulgatus* Induced by Tegoprazan Suppresses Pathogenic Bacterial Adhesion

**Figure 5:**
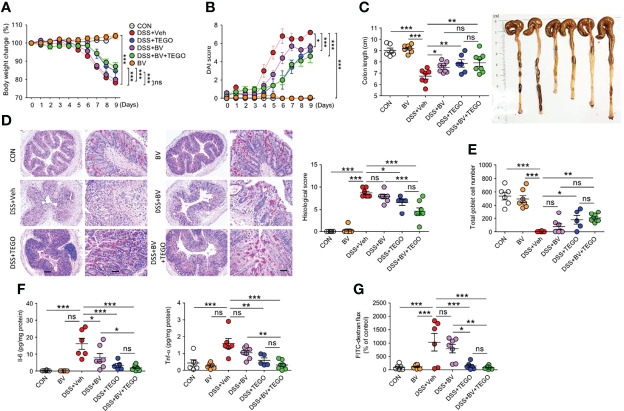
*Bacteroides vulgatus* alleviates aspects of DSS-induced colitis. Colitis was induced in 8-week-old male mice by administering 2% DSS in distilled drinking water for 5 days, followed by normal drinking water for 4 days. *B vulgatus* (5 × 10^8^ CFU/mouse/day) was administered daily and tegoprazan (30 mg/kg) and rabeprazole (30 mg/kg) were administered orally, twice daily throughout the experimental period: CON and DSS+Veh (each n = 6), DSS+TEGO (n = 5), DSS+BV and BV (n = 7), and DSS+BV+TEGO (n = 8). After sacrificing animals on Day 9, the entire colon from anesthetized mice was harvested for further investigations. The detailed methods are described in the Materials and Methods section. **(A)** Body weight change. **(B)** Disease activity index. **(C)** Representative images of colons and colon length. **(D, E)** Representative images following periodic acid-Schiff (PAS)-staining and histological score **(D)** and goblet cell number **(E)**. Scale bars = 20 μm. **(F)** Plasma level of Il-6 and Tnf-α. Plasma cytokine concentrations were measured using cytometric bead array analysis. **(G)** FITC-dextran flux (arbitrary unit) was plotted as a percentage of the control. Data represent the mean ± standard error of the mean (S.E.M). Significance is indicated by *p < 0.05, **p < 0.01, and ***p < 0.001 using two-way ANOVA followed by Bonferroni *post hoc* test **(A, B)** and one-way ANOVA followed by Dunnett’s multiple comparison test **(C–F)**. ns, not significant; CON, normal control; BV, treated with *B vulgatus*; DSS, treated with dextran sulfate sodium; Veh, treated with vehicle; TEGO, treated with tegoprazan; Il-6, interleukin-6; Tnf-α, tumor necrosis factor.

Next, to further address the impact of *B. vulgatus* on suppressing intestinal inflammation, *B. vulgatus* and tegoprazan were administered to DSS-induced colitis mice. As shown in **Figure 5**, the administration of *B. vulgatus* alone marginally attenuated DSS-induced colitis. Additionally, tegoprazan treatment and co-administration of *B. vulgatus* and tegoprazan prevented colitis in terms of weight loss, diarrhea, bleeding, colon shortening, histological damage, and goblet cell loss (**Figures 5A–E**), suggesting that the main anti-colitic effects were derived from tegoprazan. *Bacteroides vulgatus* administration drastically reduced the plasma IL-6 levels when compared with the DSS+Veh group (**Figure 5F**). The DSS+BV+TEGO group more significantly reduced plasma IL-6 and Tnf-α levels. Notably, *B. vulgatus* administration reduced gut permeability (**Figure 5G**). These data suggest that *B. vulgatus* can be implicated in the *in vivo* anti-colitic effects of tegoprazan.

**Figure 6:**
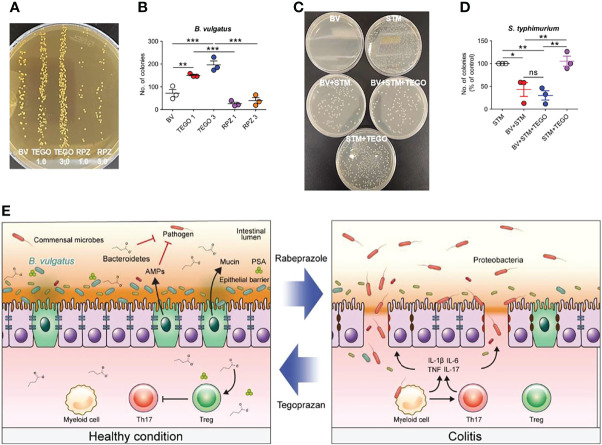
Tegoprazan inhibits epithelial adhesion of pathogenic bacteria by directly promoting the growth of *Bacteroides vulgatus*. **(A, B)** Effect of tegoprazan on the growth of *B vulgatus.* Data represent the mean ± standard error of the mean (S.E.M) of 4 independent experiments. **(A)** Representative image of *B vulgatus* growth treated with tegoprazan and rabeprazole. **(B)** Quantification of *B vulgatus* plated on GAM agar plate, plotted as the number of colonies. **(C, D)** Effect of *B vulgatus* on the attachment of pathogenic bacteria to epithelial cells. Data represent the mean ± standard error of the mean (S.E.M) of 3 independent experiments. **(C)** Representative images of adhered *Salmonella typhimurium* colonies. **(D)** Quantification of *S. typhimurium* plated on nutrient agar plate, plotted as percdentage of control. **(E)** Graphical summary. Significance is indicated by *p < 0.05, **p < 0.01, and ***p < 0.001 using one-way ANOVA followed by Dunnett’s multiple comparison test. ns, not significant; BV, *B vulgatus* only; TEGO 1, treated with 1.0 μM tegoprazan; TEGO 3, treated with 3.0 μM tegoprazan; RPZ 1, treated with 1.0 μM rabeprazole; RPZ 3, treated with 3.0 μM rabeprazole; STM, *S. Typhimurium* only, BV+STM, *S. Typhimurium* treated with *B vulgatus*; BV+STM+TEGO, *S. Typhimurium* treated with *B vulgatus* and tegoprazan; STM+TEGO, *S. Typhimurium* treated with tegoprazan; GAM, Gifu anaerobic medium.

To determine whether tegoprazan is directly involved in *B. vulgatus* growth, tegoprazan or rabeprazole was added to the *B. vulgatus* culture medium at an optical density (OD_600_) of 0.1. After 3 h, the samples were serially diluted and plated on GAM agar plates. We observed that tegoprazan promoted *B. vulgatus*, whereas rabeprazole did not promote growth (**Figures 6A, B****)**. Pathogenic bacterial adhesion on host cells plays a critical role in both inflammation and infection (27). Accordingly, a bacterial adhesion assay was performed to determine whether *B. vulgatus* affects the epithelial adhesion of pathogenic microorganisms. *Salmonella typhimurium* belongs to the Enterobacteriaceae family of the γ-Proteobacteria class and is a well-described important pathogen, similar to *E. coli*. *S. typhimurium* increases tight junction permeability, penetrates the intestinal mucosa, and triggers an acute inflammatory response (28). For inducing competition between *B. vulgatus* and *S. typhimurium* for adhesion to intestinal epithelial cells, the two bacteria were mixed in an equal volume and then added to HT-29 cells. Herein, we observed that *B. vulgatus* inhibited *S. typhimurium* adhesion to HT-29 cells (**Figures 6C, D****)**. However, tegoprazan did not prevent the adhesion of *S. typhimurium* alone, suggesting that tegoprazan impedes epithelial adhesion of pathogenic bacteria in the presence of *B. vulgatus*. Collectively, these observations indicate that tegoprazan directly promotes the growth of *B. vulgatus*, which inhibits the epithelial adhesion of pathogenic bacteria and helps relieve intestinal inflammation.

## Discussion

The intestinal mucosal barrier plays a vital role in IBD, as this is the first barrier to afford protection against foreign pathogens. Disruption of the epithelial barrier may result in microbial passage to the lamina propria, possibly eliciting an immune response. Tegoprazan is a new gastric acid inhibitor that utilizes a mechanism distinct from that of traditional PPIs to overcome the shortcomings of PPIs. Interestingly, tegoprazan improved colitis in DSS and DNBS colitis models. Tegoprazan also reduced the expression of proinflammatory cytokines, such as *Tnfa*, *Il6*, and *Il1b*. However, rabeprazole failed to demonstrate any anti-colitic effect. In addition, elevated splenic Tregs (**Supplementary Figure S1J**) suggest that immune cell modulation, at least in part, mediates the anti-colitic effect of tegoprazan. Tight junctions located in the epithelial and endothelial cell layers play essential roles in the formation of the intestinal epithelial barrier. Accordingly, disruption of the intestinal tight junction barrier increases intestinal permeability, disturbing the mucosal immune system (29). Thus, regulation of tight junctions is critical for maintaining epithelial barrier integrity in IBD. In the DSS-induced colitis model, tegoprazan reduced intestinal permeability and upregulated the expression of tight junction proteins Zo-1 and Occludin. Consistent with *in vivo* results, tegoprazan promoted the expression of tight junction proteins and reduced gut permeability in a Caco-2 cell monolayer model. In contrast, rabeprazole did not alleviate barrier damage, revealing a tendency to worsen barrier function in a dose-dependent manner. Nevertheless, we emphasize that tegoprazan affords a direct protective effect on intestinal barrier function, in contrast to rabeprazole.

Dysbiosis of the gut microbiota contributes to the pathogenesis of IBD by disrupting the intestinal mucosal barrier and inflammatory responses. Long-term use or overuse of PPIs reportedly affects microbiota composition, promotes bacterial overgrowth, and increases the risk of serious clinical outcomes of infective diseases (30). In particular, long-term PPI administration reportedly exacerbates the severity of IBD. However, it remains unclear whether dysbiosis can be attributed to the acid-suppressive effect or the specific drug-class effect of PPIs (8, 26). In the present study, metagenome analysis was performed to confirm whether tegoprazan reverts gut dysbiosis, unlike PPI, and to assess the effect of tegoprazan-induced gastric acid suppression on the intestinal microbiota composition. We revealed that tegoprazan improves microbiota diversity and enrichment in feces, while rabeprazole aggravates the dysbiosis. The anti-colitic effect of tegoprazan was attributed to a distinct mechanism of tegoprazan, but not the pH change induced by potassium channel involvement, judging from the lack of pH differences between tegoprazan and rabeprazole groups *in vitro* and *in vivo* (**Figure 4E**; **Supplementary Figure S2**). A previous study has reported that vonoprazan, another potent P-CAB, worsens nonsteroidal anti-inflammatory drug (NSAID)-induced small intestinal injury by reducing beneficial bacteria. Nadatani et al. have revealed that Bacteroidetes is rarely present in the small intestine by analyzing the small intestinal microbiota (31). One theory is that vonoprazan has an alkaline pKa of 9.3 (32), and accordingly shows relatively weak action in the stomach but presents higher activity in the alkaline small intestine, which might alter the intestinal microbiota. In contrast, tegoprazan has a pKa of 5.2, which is only activated in the stomach and has insignificant effect on the small intestine. Therefore, it can be considered that this factor minimally impacts changes in the intestinal microbiota. In addition, the NSAID-induced small intestinal injury model and the DSS- and DNBS-induced colitis models have different mechanisms of action (33), and the composition of microbiota markedly differ in the small intestine and colon (34); this may explain the discrepancies observed. However, as the anti-inflammatory effects of other P-CAB drugs remain elusive, further research is required to determine whether the anti-colitic effect of tegoprazan is a specific drug-class effect of P-CABs. Moreover, we cannot exclude the potential intermediary role of tegoprazan in immune cells such as Tregs.

Herein, tegoprazan drastically increased the levels of Bacteroidetes and decreased those of Proteobacteria—which had previously been reduced and elevated by DSS, respectively—at the phylum level. In contrast, rabeprazole did not enhance the abundance of Bacteroidetes but markedly increased that of Proteobacteria. Rabeprazole significantly increased the abundance of *E. coli*, which belongs to the phylum Proteobacteria (**Figures 4C, D****)**. Notably, spontaneous colitis induction in toll-like receptor (TLR)-5 and IL-10 knockout mice has been associated with an expansion of *Proteobacteria* (35, 36). Indeed, Proteobacteria are often found to be elevated in IBD (37, 38), and a higher level of Proteobacteria has been detected in patients with severe IBD. Based on these results, we confirmed that rabeprazole exacerbated colitis by abnormally expanding Proteobacteria. Interestingly, our experiments indicate that Bacteroidetes, a counterpart of abundant obligate anaerobes in the normal intestine, such as *B. vulgatus*, plays a pivotal role in protecting against colitis beyond Firmicutes. Conversely, dysbiosis can be characterized by an imbalance between obligate and facultative anaerobes, termed “oxygen hypothesis” (39). In consequence, inflammation elicits a favorable environment for γ-proteobacteria owing to their relatively higher tolerance for oxygen diffused from the epithelium.

Approximately 25% of the commensal bacteria in the healthy human colon are species of *Bacteroides*, the most predominant anaerobes (40), with phylogenetic proximity detected between mouse and human *B. vulgatus* strains in the gut (41). We noted that *B. vulgatus* is related to the suppression of pathogenic Proteobacteria, in addition to the well-known modulation of the inflammatory response, such as the inhibition of Th17-related cytokines by polysaccharide A (42), intestinal-associated lymphoid tissue development (43), or enhancing barrier function through IL-10 production and reducing proinflammatory NF-κB signaling (44). Several remote studies have revealed that *B. vulgatus* is associated with gut inflammation (45–48), whereas recent studies have reported that *B. vulgatus* has a protective effect on intestinal inflammation. In IL-2 knockout mice, *B. vulgatus* reportedly ameliorates *E. coli*-induced colitis development (49). Moreover, the administration of *B. vulgatus* has been shown to reduce LPS production by gut microbiota and protect against atherosclerosis in a coronary artery disease mouse model (50). *Bacteroides vulgatus* also inhibited intestinal infections by *Vibrio cholera* (51). Propionic and butyric acids, which suppress Proteobacteria, are substantially dampened by *B. vulgatus* elimination (51). Intriguingly, this study revealed that tegoprazan increased the abundance of *B. vulgatus*. Indeed, bacterial growth experiments revealed that tegoprazan directly promoted the growth of *B. vulgatus*. However, rabeprazole did not promote *B. vulgatus* growth. Adhesion of pathogenic bacteria, such as Proteobacteria, to host epithelia is crucial, as passage of bacteria across epithelial cells and entry into the lumen leads to infection (52). In the bacterial adhesion assay, *B. vulgatus* inhibited the adhesion of *S. typhimurium* to the epithelial cells; however, tegoprazan alone did not affect *S. typhimurium* adhesion to the epithelial cells. These results reveal that tegoprazan ameliorates intestinal inflammation by increasing the abundance of *B. vulgatus*, and that increased levels of *B. vulgatus* play a marginal role in preventing intestinal invasion by blocking the adhesion of pathogenic bacteria (**Figure 6E**). Polysaccharide A, produced by *B. fragilis*, reportedly promotes the differentiation of functional Tregs (53), and Tregs, produced by Bacteroidetes, play an important role in maintaining mucosal tolerance (54). Accordingly, Treg induction by tegoprazan administration is considered another anti-inflammatory mechanism. Therefore, further investigations are needed to clarify the mechanism through which tegoprazan promotes *B. vulgatus* and Tregs, as well as to determine whether it improves intestinal inflammation in patients with IBD. This is the first study to explain how tegoprazan regulates colitis and presents a link between tegoprazan and gut microbiota. Additionally, we revealed that *B. vulgatus* was involved in mediating the anti-colitic effect of tegoprazan, suggesting that tegoprazan may be useful in microbiota control and as a novel protective agent against IBD.

In conclusion, tegoprazan, a novel P-CAB, exhibits stronger and more reversible gastric acid suppression than traditional PPIs. However, the effect of tegoprazan on intestinal inflammation remains unknown. Long-term PPI administration reportedly increases and exacerbates the severity of IBD. To our knowledge, this study is the first to demonstrate that tegoprazan potentially ameliorates intestinal inflammation by enhancing intestinal epithelial barrier integrity, increasing the levels of Tregs, and modulating the composition of the gut microbiota, as demonstrated by *in vitro* and *in vivo* experiments. Additionally, we determined that tegoprazan promotes the growth of a particular member of commensal bacteria, *B. vulgatus*, which crucially contributes to the suppression of pathogenic microorganisms. Specifically, our findings provide critical insights into the potential treatment strategies using PPIs and P-CABs in IBD, although detailed mechanisms underlying the changes described warrant further characterization.

## Data Availability Statement

The original contributions presented in the study are included in the article/**Supplementary Material**, further inquiries can be directed to the corresponding authors.

## Ethics Statement

The animal study was reviewed and approved by Institutional Animal Care and Use Committee of Yonsei University.

## Author Contributions

Conceptualization: SWK, MS, JH, and JHC. Methodology: MS, ISP, SK, HWM, and JHK. Investigation: MS, ISP, and SWK. Visualization: MS, ISP, and SWK. Supervision: JHC and SWK. Writing—original draft: MS, SWK, and JHC. Writing—review & editing: TIK and WHK. All authors contributed to the article and approved the submitted version.

## Funding

This study received funding from the HK INNO.N corporation (2018–31–0929). The funder was not involved in the study design, collection, analysis, interpretation of data, the writing of this article or the decision to submit it for publication. This work was also supported by the Brain Korea 21 Project for Medical Science, Yonsei University.

## Conflict of Interest

The authors declare that the research was conducted in the absence of any commercial or financial relationships that could be construed as a potential conflict of interest.

## Publisher’s Note

All claims expressed in this article are solely those of the authors and do not necessarily represent those of their affiliated organizations, or those of the publisher, the editors and the reviewers. Any product that may be evaluated in this article, or claim that may be made by its manufacturer, is not guaranteed or endorsed by the publisher.
