# Supplementary material for: Novel Potassium-Competitive Acid Blocker, Tegoprazan, Protects Against Colitis by Improving Gut Barrier Function
Source: Front Immunol. 2022 May 25;13:870817. doi: 10.3389/fimmu.2022.870817 (PMC9174989; doi:10.3389/fimmu.2022.870817)
Supplement: Supplementary file 1 [file DataSheet_1.pdf]

Supplementary Materials for

Novel potassium-competitive acid blocker, tegoprazan, protects against colitis

Mijeong Son, I Seul Park, Soochan Kim, Hyun Woo Ma, Ji Hyung Kim, Tae Il Kim, Won Ho Kim, Jaeyong Han, Seung Won Kim, Jae Hee Cheon

*Corresponding author. Email: GENIUSHEE@yuhs.ac, swk21c@hanmail.net, md.hanj@gmail.com

This PDF file includes:

Supplementary Text
Figs. S1 to S4
Tables S1 to S3

Supplementary Text

Supplementary Methods

Dinitrobenzene sulfonic acid (DNBS)-induced colitis model

DNBS-induced colitis was induced by rectally administering DNBS (556971, Sigma-Aldrich, St. Louis, MO, USA). Mice were fasted for 24 h before colitis induction and lightly anesthetized by isoflurane inhalation. Then, a polyethylene catheter was inserted into the rectum, and 5 mg of DNBS dissolved in 100 µL of 50% ethanol was administered into the colon. The control group was administered 50% ethanol. Mice were randomly divided into four groups: 1) control group (CON), 2) DNBS + vehicle group (DNBS+Veh), 3) DNBS + tegoprazan treated group (DNBS+TEGO), and 4) DNBS + rabeprazole-treated group (DNBS+RPZ). Tegoprazan (30 mg/kg) and rabeprazole (30 mg/kg) were administered orally, twice daily throughout the experimental period. All drugs were kindly provided by HK INNO.N corporation and were dissolved in 0.5% (w/v) methylcellulose (Sigma-Aldrich). On day 5, the entire colon was harvested by performing an autopsy for further analysis.

Dextran sulfate sodium (DSS)-induced colitis model

In experiment using filgotinib, experimental ulcerative colitis was induced by administering 2.5% (w/v) DSS (molecular weight: 36–50 kDa, 160110, MP Biomedicals, Santa Ana, CA, USA) in distilled drinking water for 5 days, followed by normal drinking water for 4 days. Mice were randomly divided into four groups: 1) control group (CON), 2) DSS+vehicle group (DSS+Veh), 3) DSS+tegoprazan-treated group (DSS+TEGO), and 4) DSS+filgotinib-treated group (DSS+FILGO). The control group received normal drinking water without DSS during the entire experimental period. Tegoprazan and filgotinib (30 mg/kg) were administered orally daily throughout the experimental period q.d. and b.d., respectively. All drugs were dissolved in 0.5% (w/v) methylcellulose (Sigma-Aldrich). A daily assessment of body weight and disease activity was performed for each mouse. On day 9, the entire colon and blood were harvested by autopsy for further investigations.

In experiment using rabeprazole, colitis was induced by administering 2% (w/v) DSS in distilled drinking water for 5 days, followed by normal drinking water until the end of the experiment. Mice were randomly divided into four groups: 1) control group (CON), 2) DSS+vehicle group (DSS+Veh), 3) DSS+tegoprazan-treated group (DSS+TEGO), and 4) DSS+rabeprazole-treated group (DSS+RPZ). The control group received normal drinking water without DSS during the entire experimental period. Tegoprazan (30 mg/kg) and rabeprazole (30 mg/kg) were administered orally, twice daily throughout the experimental period. A daily assessment of body weight and disease activity was performed for each mouse. All drugs were dissolved in 0.5% (w/v) methylcellulose. On day 9, blood was collected from the hearts of anesthetized mice, and the entire colon and blood were harvested by autopsy for further investigations. The blood was collected into 2 mL tubes containing EDTA and centrifuged at 1500 g for 15 min. Next, the plasma phase above buffy coat was collected and stored at -70°C before use.

To further determine whether *B. vulgatus* can attenuate the severity of colitis, experimental colitis was induced by DSS, as previously described. The mice were randomly divided into six groups: 1) control group (CON), 2) *B. vulgatus group* (BV), 3) DSS + vehicle group (DSS + Veh), 4) DSS + *B. vulgatus* group (DSS+BV), 5) DSS+tegoprazan group (DSS+TEGO), and 6) DSS + *B. vulgatus* + tegoprazan group (DSS+BV+TEGO). *B. vulgatus* (5

× 10^8 colony-forming units (CFU)/mouse/day) and tegoprazan (30 mg/kg BID) were administered by oral gavage until the end of the experiment.

Disease activity index (DAI)

Mice were assessed daily for colitis development by monitoring weight loss, rectal bleeding, and stool consistency (summarized in Table S1). The percentage of body weight loss was calculated relative to the initial BW (Day 0) using the following formula: [(weight on day X-Initial weight)/initial weight] × 100.

Extraction of RNA and quantitative real-time reverse-transcription polymerase chain reaction

Total RNA was extracted using TRIzol Reagent (Thermo Fisher Scientific, Cambridge, MA, USA) and the Ribospin™ total RNA purification kit (304-150, GeneAll Biotechnology, Seoul, Korea). Then, RNA (1 μg) was reverse-transcribed using a High Capacity cDNA Reverse Transcription Kit (4368813, Thermo Fisher Scientific) according to the manufacturer's protocol. Amplification was performed using Power SYBR green (4367659, Thermo Fisher Scientific) and StepOne Plus real-time PCR system (Thermo Fisher Scientific) for 45 cycles using the following thermocycling steps: 95°C for 30 s, 59-61°C for 30 s, and 72°C for 40 s. Gene expression levels were calculated after normalization to the standard housekeeping gene *β-actin* using the relative comparative method with the following equation: relative gene expression = $2^{-(\Delta Ct\ sample - \Delta Ct\ control)}$. Results were reported as the fold change when compared with the calibrator or $2^{\Delta Ct}$ after normalization of the transcript level to the average of the endogenous control. The primers used for the real-time PCR are listed in Table S3.

Immunohistochemistry (IHC)

Formalin-fixed, paraffin-embedded colon sections were deparaffinized in xylene and ethanol and rehydrated in water. Antigen retrieval was performed in a heated sodium citrate buffer (pH 6.0) for 10 min. Sections were washed with distilled water, quenched in 0.3% hydrogen peroxide to block endogenous peroxidase activity, and blocked in 3% bovine serum albumin (BSA) diluted in Tris-buffered saline plus 0.1% Tween-20 (TBS-T) for 30 min at room temperature. Then, sections were incubated with anti-Zo-1 antibody (1:500, ab59720, Thermo Fisher Scientific) diluted in blocking buffer overnight at 4°C, following which biotinylated anti-rabbit secondary antibody (1:500, BP-9100-50, Vector Laboratories, Burlingame, CA, USA) was applied for 30 min at room temperature. VECTASTAIN® Elite ABC-HRP Kit (PK-6100, Vector Laboratories) was employed, and staining was visualized using the DAB Substrate Kit (SK-4100, Vector Laboratories). Slides were counterstained with hematoxylin, dehydrated, mounted, and observed under a microscope. qRT-PCR, western blotting, and flow cytometric analysis are described in the Supporting Information.

Metagenomic analysis of the microbiome

Bacterial genomic DNA was obtained from fecal and tissue samples using SPIN for Soil Kit (116560200, MP Biomedicals), according to the manufacturer's instructions. Extracted DNA was amplified using bar-coded primers flanking the V3-V4 region of the 16S rRNA gene by ChunLab Inc. (Seoul, Korea). For bacterial amplification, fusion primers of 341F (5′-AATGATACGGCGACCACCGAGATCTACAC-XXXXXXXX-TCGTCGGCAGCGTC-AGATGTGTATAAGAGACAG-<u>CCTACGGGNGGCWGCAG</u>-3′; underlining sequence

indicates the target region primer) and 805R (5′- CAAGCAGAAGACGGCATACGAGAT-XXXXXXXX-GTCTCGTGGGCTCGG-AGATGTGTATAAGAGACAG-GACTACHVGGGTATCTAATCC-3′). The Fusion primers are constructed in the following order which is P5 (P7) graft binding, i5 (i7) index, Nextera consensus, Sequencing adaptor, and Target region sequence. The amplifications were carried out under the following conditions: initial denaturation at 95°C for 3 min, followed by 25 cycles of denaturation at 95°C for 30 s, primer annealing at 55°C for 30 s, and extension at 72 °C for 30 s, with a final elongation at 72°C for 5 min. The PCR product was analyzed by using 1% agarose gel electrophoresis and visualized under a Gel Doc system (BioRad, Hercules, CA, USA). The amplified products were purified with the CleanPCR (CleanNA). Equal concentrations of purified products were pooled together and short fragments (non-target products) were removed with CleanPCR (CleanNA, Waddinxveen, The Netherlands). The quality and product size were assessed on a Bioanalyzer 2100 system (Agilent, Palo Alto, CA, USA) using a DNA 7500 chip. Mixed amplicons were pooled and the sequencing was carried out at Chunlab, Inc., with Illumina MiSeq Sequencing system (Illumina, CA, USA) according to the manufacturer's instructions.

Reads obtained from different samples were sorted by unique barcodes of each PCR product. Processing raw reads start with quality check and filtering of low quality (<Q25) reads by Trimmomatic 0.32 (*52*). After QC pass, paired-end sequence data are merged using PANDAseq (*53*). Primers are then trimmed with ChunLab's in-house program at a similarity cut-off of 0.8. Non-specific amplicons that do not encode 16S rRNA are detected by HMMER's hmmsearch program (*54*) with 16S rRNA profiles. Sequences are denoised using DUDE-Seq (*55*) and non-redundant reads are extracted by UCLUST-clustering (*56*). The EzBioCloud database is used for taxonomic assignment using USEARCH (8.1.1861_i86linux32) (*56*) followed by more precise pairwise alignment (*57*). UCHIME (*58*) and the non-chimeric 16S rRNA database from EzBioCloud are used to detect chimera on reads with <97% similarity. Reads that are not identified to the species level (with <97% similarity) in the EzBioCloud database are compiled and UCLUST (*56*) and CDHIT (*59*) are used to perform de-novo clustering to generate additional OTUs. Finally, OTUs with single reads (singletons) are omitted from further analysis. The alpha diversity indices (*60*) are estimated by in-house code. Microbiome data were analyzed with the 16S-based microbial taxonomic profiling (MTP) platform of EzBio-Cloud (ChunLab Inc.). All 16S rRNA sequences were deposited in the ChunLab EzBioCloud Microbiome Database (*63*). Herein, we utilized the eztaxon-e taxonomic structure (*64*) as the backbone phylogenetic tree. Both UniFrac and Fast Unifrac algorithms were developed by Knight and colleagues (65). We analyzed operational taxonomic units and beta diversity using principal coordinate analysis (PCoA) and alpha diversity analysis using diversity indices (Chao1, Ace, Shannon, and Simpson indices). Permutational multivariate analysis of variance (PERMANOVA) was used to analyze significant differences in alpha diversity. Bacterial community abundance was determined using CLcommunity (ChunLab Inc.).

Immunofluorescence analysis

Caco-2 cells were fixed with 10% neutral buffered formalin for 30 min at room temperature and washed with phosphate-buffered saline (PBS). Next, cells were blocked with 5% BSA for 1 h at room temperature and incubated with rabbit anti-E-cadherin antibody (1:200, Cell Signaling Technology, Inc., Beverly, MA, USA) diluted in blocking buffer overnight at 4°C. Cells were then washed with PBS, incubated with the secondary antibody, Alexa Fluor 488 goat anti-rabbit IgG H&L (1:200, A-11008, Invitrogen, Cambridge, UK) for 1 h at room temperature, and

nuclear stained with diamidino-2-phenylindole (DAPI, 300 nM, D1306, Thermo Fisher Scientific) for 3 min. Confocal images were obtained using a Zeiss LSM700 confocal microscope (Carl Zeiss AG, Oberkochen, Germany).

Western blotting

Caco-2 cells were lysed with Pierce RIPA buffer (Thermo Fisher Scientific, Rockford, IL, USA) supplemented with Halt protease and phosphatase inhibitor cocktail (Thermo Fisher Scientific). Protein concentration was measured with Pierce™ BCA Protein Assay Kit (Thermo Fisher Scientific, Waltham, Massachusetts, USA). Proteins were separated according to their molecular weight by sodium dodecyl sulfate (SDS)-polyacrylamide gel electrophoresis and subsequently transferred to polyvinylidene fluoride membranes.

Membranes were blocked for 1 h with TBS-T supplemented with 5% skimmed milk and then incubated with anti-ZO-1 (1:1,000, Thermo Fisher Scientific), anti-E-cadherin (1:1,000, Cell Signaling Technology, Inc.), and anti-β-actin (1:5000, Sigma-Aldrich, St Louis, MO, USA) overnight at 4°C. Then, membranes were washed and incubated with HRP-conjugated secondary antibodies (anti-rabbit, 1:2,500; anti-mouse, 1:2,500) for 1 h at room temperature. For the detection of the protein bands, ECL™ Prime western blotting Detection Reagent (Amersham, Buckinghamshire, UK) was used, and signals were imaged using a LAS 4,000 mini Biomolecular Imager (Fujifilm, Tokyo, Japan).

Bacterial adhesion assay

HT-29 cells were plated in 12-well plates in fresh medium supplemented with 10% FBS. The cells were treated with *B. vulgatus* and *S. typhimurium* for 3 h at a ratio of 50:1. Cells were then washed and removed from plates and exposed to 100 μL of 0.1% Triton-X 100 for 10 min. Next, 900 μL of nutrient broth (Difco, Becton & Dickinson, Sparks, MD, USA) was added to the plates, and the cell lysates were resuspended. Finally, cell lysates were serially diluted and plated on nutrient agar plates with 100 μg/ml ampicillin to enumerate the CFUs of the adhered bacteria.

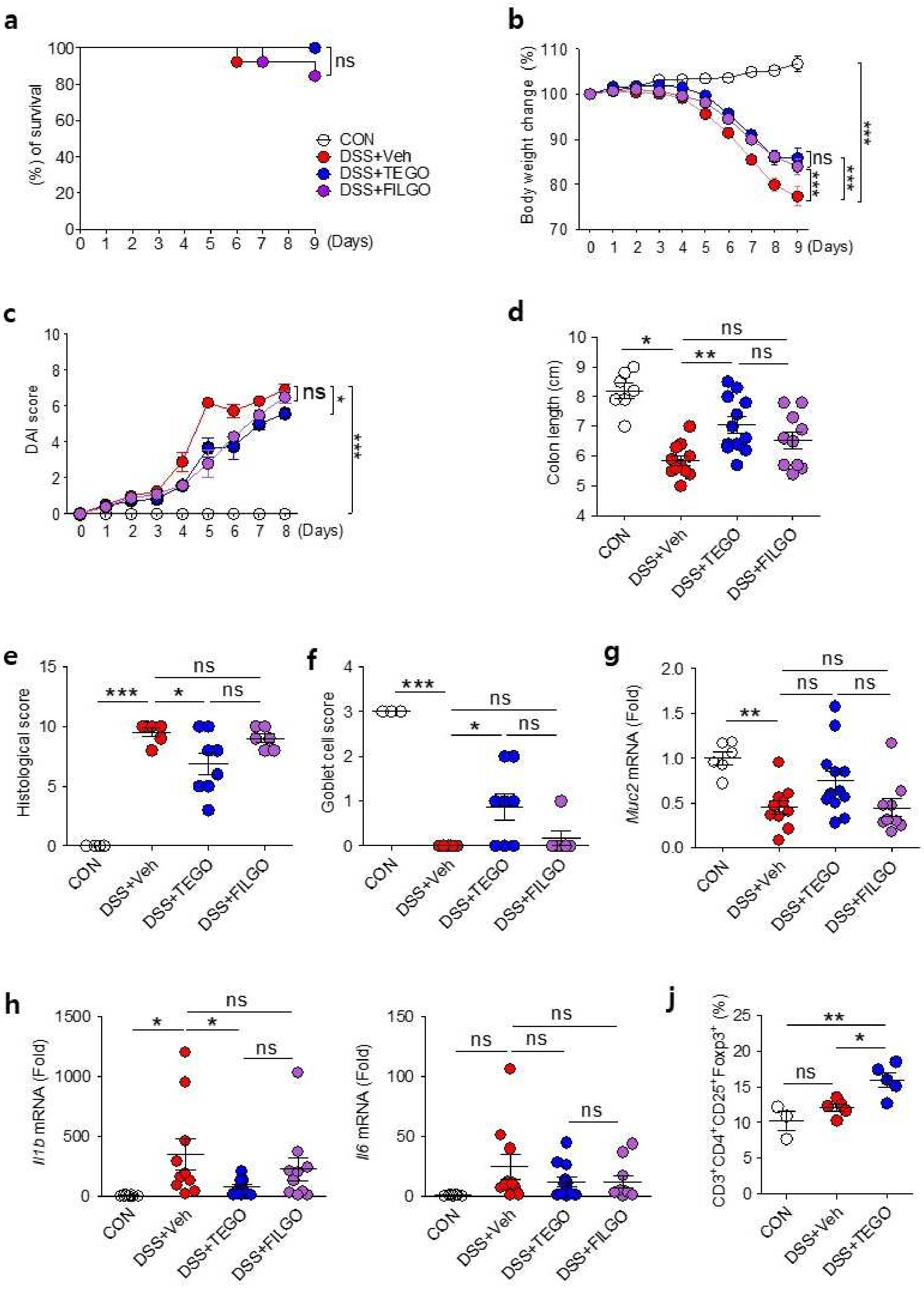
a
(%) of survival
ns
(Days)
CON
DSS+Veh
DSS+TEGO
DSS+FILGO
b
Body weight change (%)
(Days)
ns
***
c
DAI score
(Days)
ns
*
***
d
Colon length (cm)
CON
DSS+Veh
DSS+TEGO
DSS+FILGO
e
Histological score
f
Goblet cell score
g
Muc2 mRNA (Fold)
h
Il1b mRNA (Fold)
Il6 mRNA (Fold)
j
CD3+CD4+CD25+Foxp3+ (%)

i

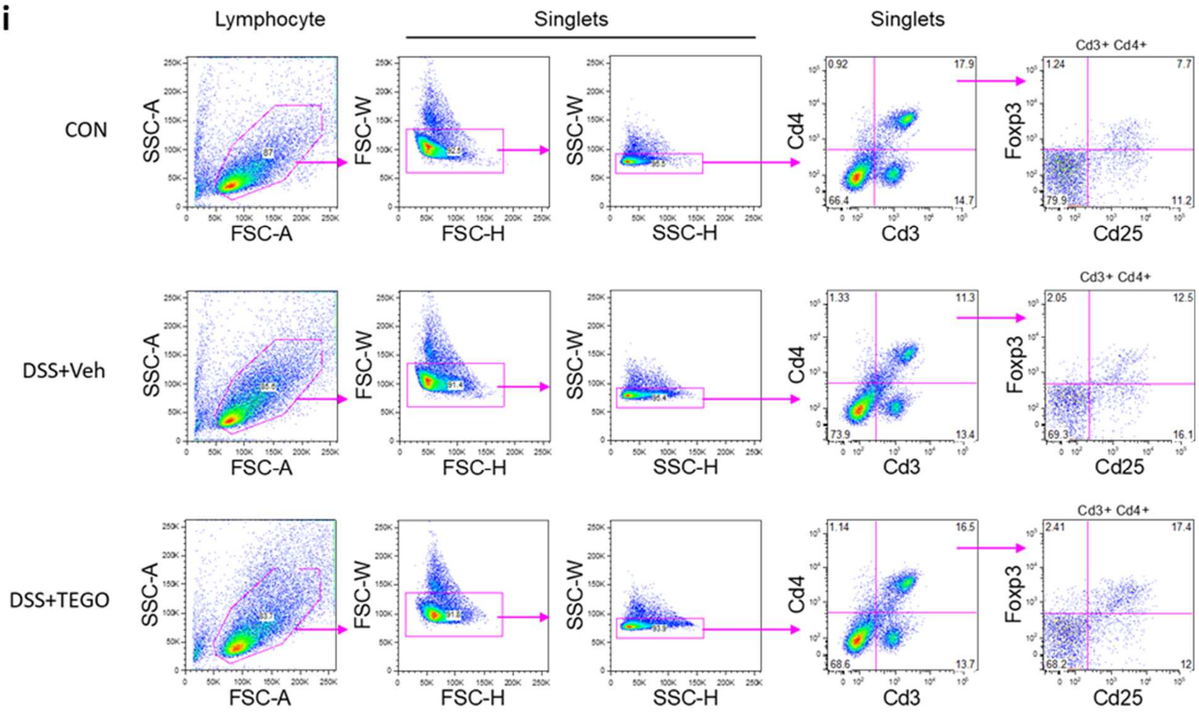

Figure S1.

Tegoprazan action is beyond the effect of filgotinib. Colitis was induced in 8-week-old male mice by administering 2.5% DSS in distilled drinking water for 5 days, followed by normal drinking water for 4 days. Tegoprazan and filgotinib (30 mg/kg) were administered orally daily throughout the experimental period q.d. and b.d., respectively. All drugs were dissolved in 0.5% (w/v) methylcellulose. **a**, Survival rate from survival model. CON (n = 7), DSS+Veh, DSS+TEGO, and DSS+FILGO (each n = 13). Survival analyses were performed using Kaplan-Meier plots for overall survival and differences were compared using a log-rank test. (**b–h**) Efficacy model. CON (n = 7), DSS+Veh (n = 11), DSS+TEGO (n = 12), and DSS+FILGO (n = 10). **b**, Body weight change. **c**, Disease activity index (DAI). **d**, Colon lengths. **e**, Histological score. **f**, Goblet cell score. **g**, Gene expression level of mucin (*Muc2*). **h**, Gene expression level of proinflammatory cytokines (*Tnfa*, *Il1b*, and *Il6*). (**i**,**j**) Flow cytometric analysis of Tregs in mouse splenocytes treated with DSS and tegoprazan. Splenocytes were isolated, stained with

antibodies, and analyzed using flow cytometry for Tregs ($CD3^{+}CD4^{+}CD25^{+}Foxp3^{+}$): CON (n = 3), DSS+Veh (n = 5), DSS+TEGO (n = 5). (**i**) Representative flow cytometry gating strategy. (**j**) We observed that Tregs ($CD3^{+}CD4^{+}CD25^{+}Foxp3^{+}$ cells) were significantly induced in the DSS+TEGO group when compared with the DSS+Veh group, indicating that tegoprazan facilitates Treg induction. Data represent the mean ± standard error of the mean (S.E.M). Significance is indicated by $*p < 0.05$, $**p < 0.01$, and $***p < 0.001$ using Student *t*-test, two-way ANOVA, or one-way ANOVA followed by Bonferroni post hoc test (**a**,**b**), or Dunnett's multiple comparison test (**c**–**j**)**.** ns, not significant; CON, normal control; DSS, treated with dextran sulfate sodium; Veh, treated with vehicle; TEGO, treated with tegoprazan; FILGO, treated with filgotinib.

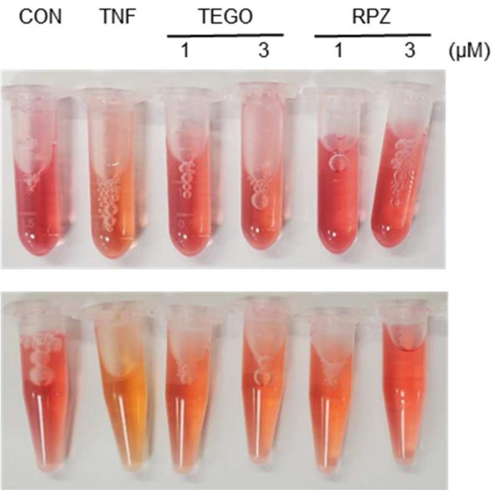

Figure S2.

Acid blocking effect by tegoprazan is not responsible for intestinal epithelial barrier function. Caco-2 cell monolayers in transwell plate or culture plate were treated with TNF-α (40 ng/mL), with or without tegoprazan or rabeprazole (1 and 3 μM) for 48 h, to disrupt the epithelial barrier. The culture media were harvested at 48 h, and images in 2 mL tubes were obtained**.** ns, not significant; CON, treated with dimethyl sulfoxide (DMSO); TNF, treated with 40 ng/mL tumor necrosis factor-α; TEGO 1, treated with 40 ng/mL TNF- α and 1.0 μM tegoprazan; TEGO 3, treated with 40 ng/mL TNF- α and 3.0 μM tegoprazan; RPZ 1, treated with 40 ng/mL TNF- α and 1.0 μM rabeprazole; RPZ 3, treated with 40 ng/mL TNF- α and 3.0 μM rabeprazole.

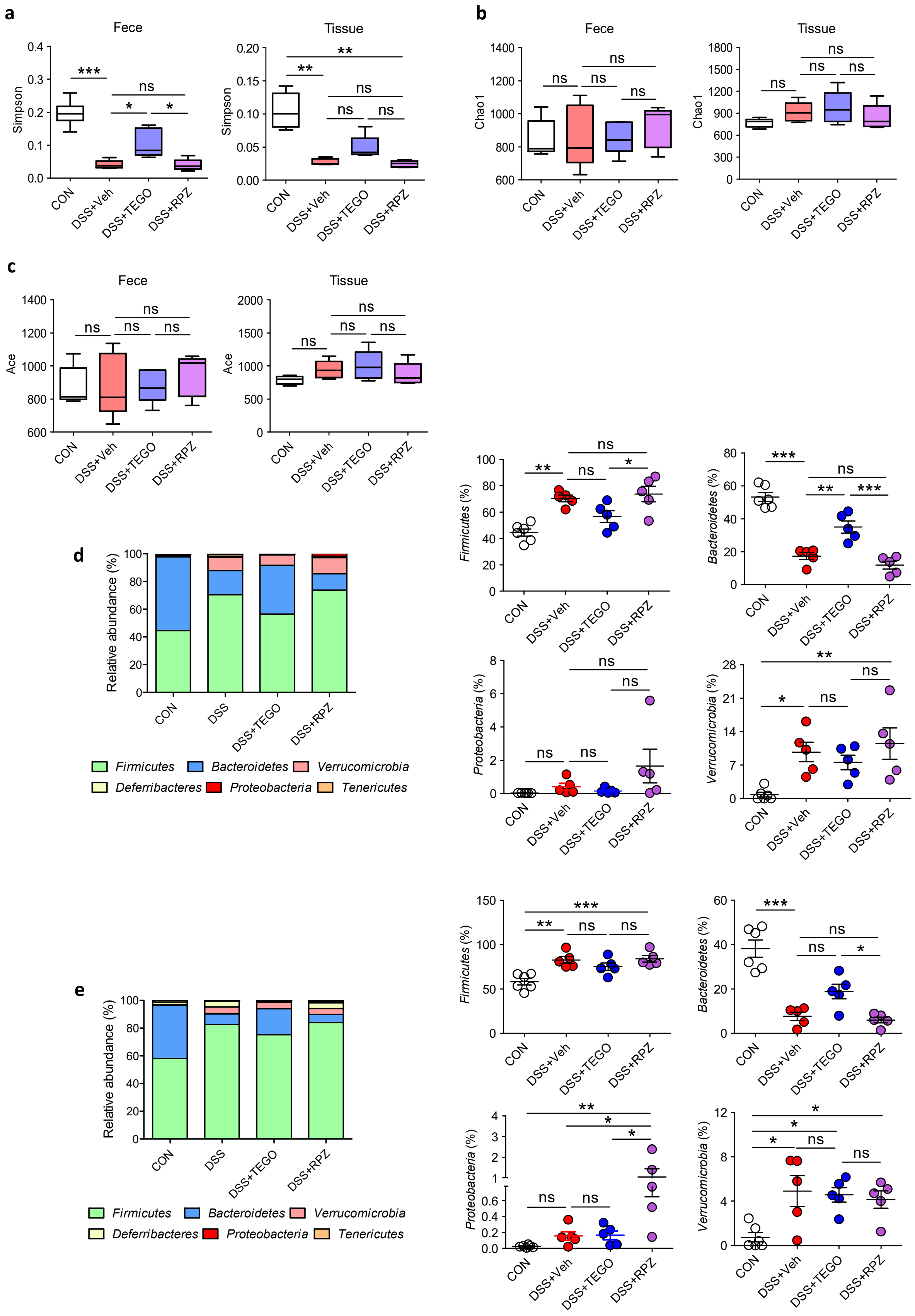
a
Fece
Tissue
Simpson
b
Fece
Tissue
Chao1
c
Fece
Tissue
Ace
CON
DSS+Veh
DSS+TEGO
DSS+RPZ
ns
d
Relative abundance (%)
CON
DSS
DSS+TEGO
DSS+RPZ
Firmicutes
Bacteroidetes
Verrucomicrobia
Deferribacteres
Proteobacteria
Tenericutes
Firmicutes (%)
Bacteroidetes (%)
Proteobacteria (%)
Verrucomicrobia (%)
e
Relative abundance (%)
Firmicutes
Bacteroidetes
Verrucomicrobia
Deferribacteres
Proteobacteria
Tenericutes

Figure S3.

Tegoprazan alters microbiota and prevents dysbiosis in colitis. Gut microbiome composition in feces and tissue was investigated using 16S rRNA sequencing: CON (n = 6), DSS+Veh, DSS+TEGO and DSS+RPZ (each n = 5). (**a–c**) Effect of tegoprazan on alpha diversity (**a**,**b**) indices in DSS-induced colitis. **a**, Simpson index. **b**, Chao1 index. **c**, Ace index. Box plots with median, $25^{th}/75^{th}$ percentiles and whiskers from minimum to maximum, and individual values plotted. (**d**,**e**) Microbial community bar plot by phylum in fece (**d**) and tissue (**e**) samples and the abundance ratio of Firmicutes, Bacteroidetes, Proteobacteria, and Verrucomicrobia. Data represent the mean or mean ± standard error of the mean (S.E.M). Significance is indicated by $^{*}p < 0.05$, $^{**}p < 0.01$, and $^{***}p < 0.001$ using one-way ANOVA followed by Dunnett's multiple comparison test. ns, not significant; CON, normal control; DSS, treated with dextran sulfate sodium; Veh, treated with vehicle; TEGO, treated with tegoprazan; RPZ, treated with rabeprazole.

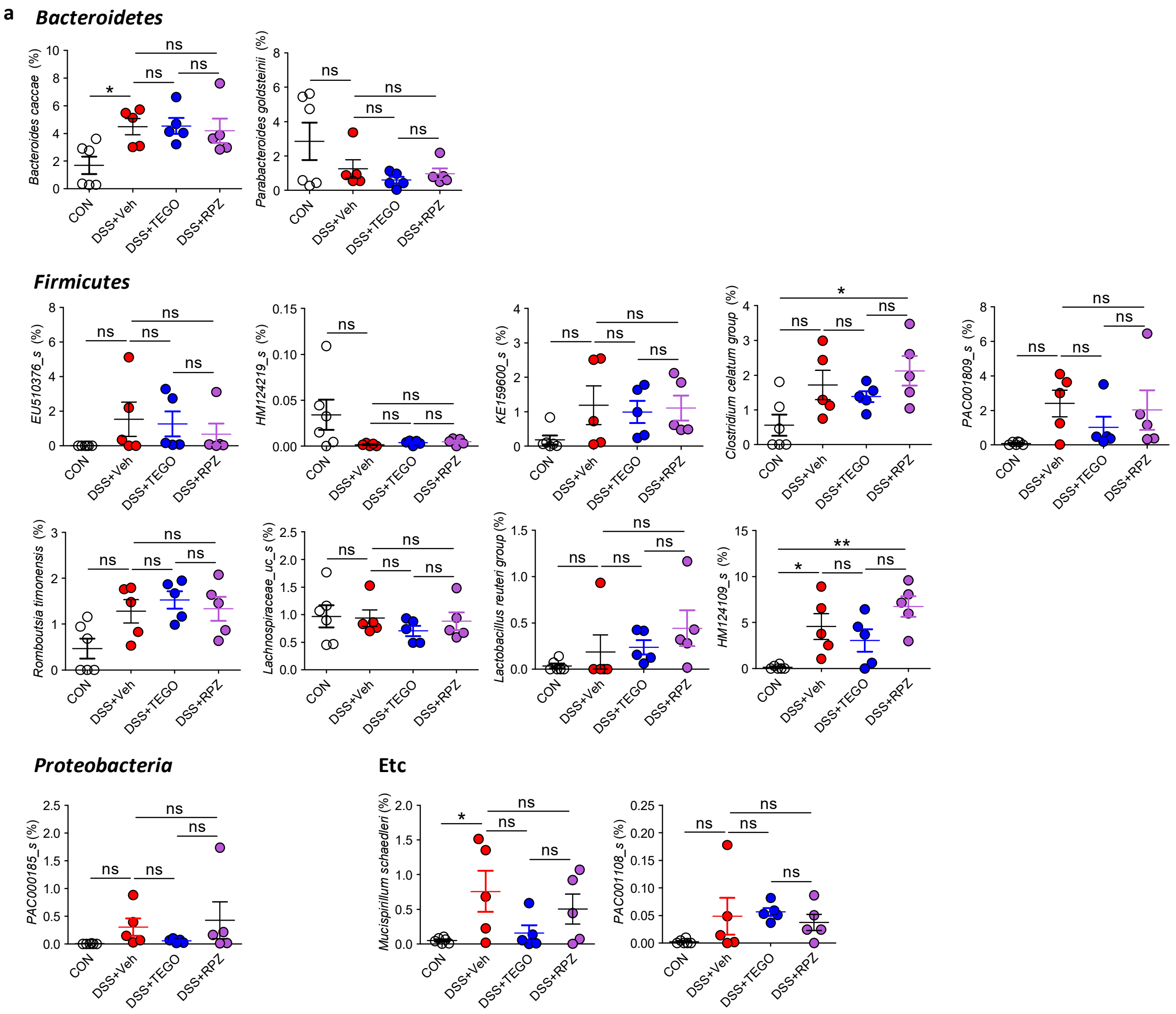
a
Bacteroidetes
Bacteroides caccae (%)
Parabacteroides goldsteinii (%)
Firmicutes
EU510376_s (%)
HM124219_s (%)
KE159600_s (%)
Clostridium celatum group (%)
PAC001809_s (%)
Romboutsia timonensis (%)
Lachnospiraceae_uc_s (%)
Lactobacillus reuteri group (%)
HM124109_s (%)
Proteobacteria
PAC000185_s (%)
Etc
Mucispirillum schaedleri (%)
PAC001108_s (%)
CON
DSS+Veh
DSS+TEGO
DSS+RPZ
ns
*
**

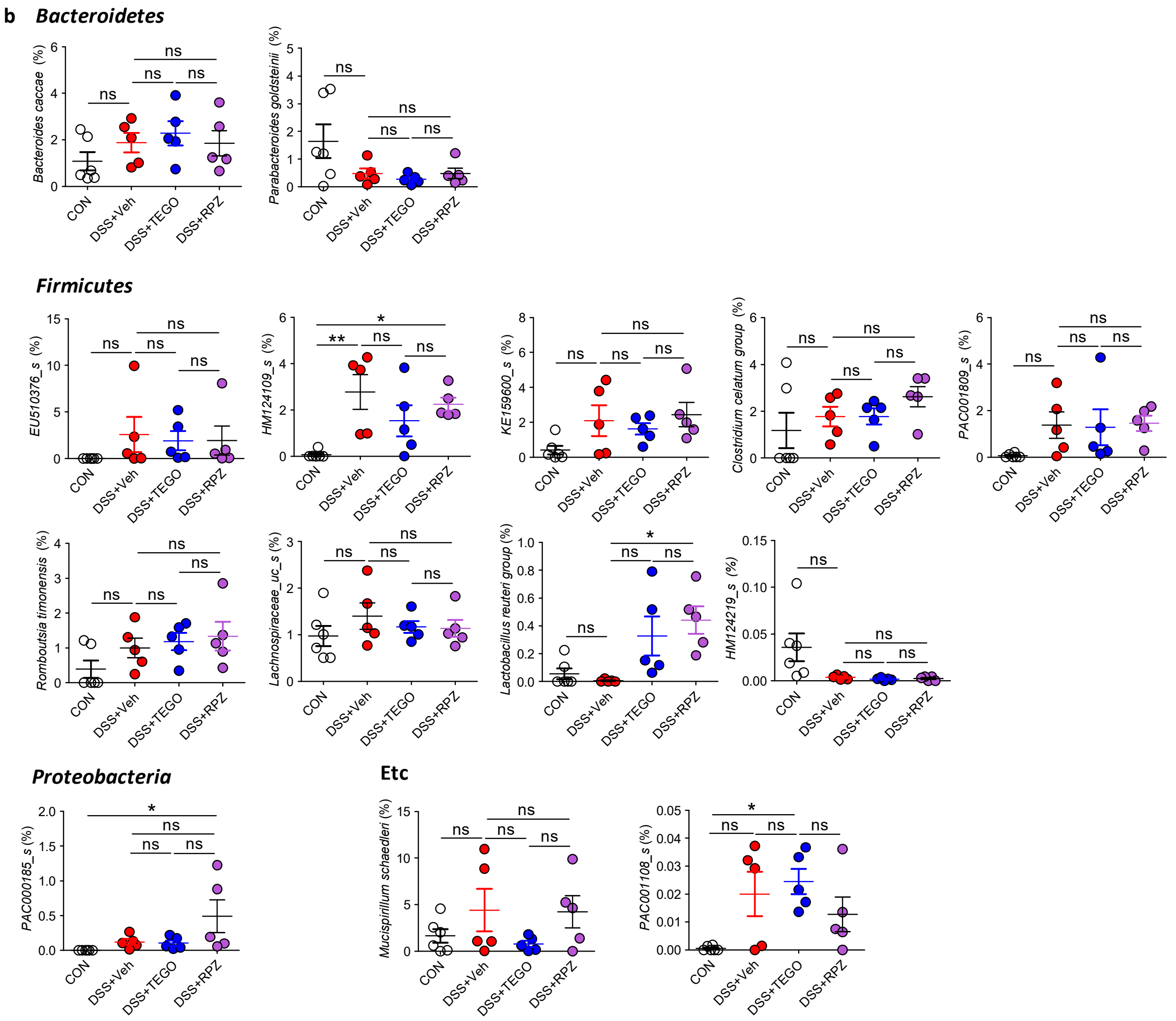

Figure S4.

Representative bacterial species belonging to phylum *Bacteroidetes*, *Firmicutes*, *Proteobacteria*, and others. Representative microbial community bar plot by species in fecal (**a**) and tissue (**b**) samples and the abundance ratio of bacteria belonging to phylum *Bacteroidetes*, *Firmicutes*, *Proteobacteria*, and others. CON (n = 6), DSS+Veh, DSS+TEGO and DSS+RPZ (each n = 5). Significance is indicated by *$p < 0.05$ and **$p < 0.01$ using one-way ANOVA followed by Dunnett's multiple comparison test. ns, not significant; CON, normal control; DSS, treated with dextran sulfate sodium; Veh, treated with vehicle; TEGO, treated with tegoprazan; RPZ, treated with rabeprazole.

Table S1.

Disease activity index (DAI) scoring system

Score	Body weight loss (%)	Stool consistency	Rectal bleeding
0	0-1 %	Normal	Normal
1	1-5 %	Moist and sticky	Visible blood
2	5-10 %	Soft	Slight bleeding
3	10-20 %	Diarrhea	Gross bleeding
4	>20 %	-	-

Table S2.
Histological scoring system

Score	Severity of inflammation	Extent of injury	Crypt damage
0	None	None	None
1	Slight	Mucosal	Basal 1/3 damaged
2	Moderate	Submucosal	Basal 2/3 damaged
3	Severe	Transmural	Only surface epithelium intact
4	-	-	Entire crypt and epithelium lost

Table S3.
Primers used for real-time PCR

Gene	Sequence (5′–3′)
Mouse	
Tnfa	F: CAAAGGGAGAGTGGTCAGGT<br>R: ATTGCACCTCAGGGAAGAGT
Il1b	F: GCAACTGTTCCTGAACTCAACT<br>R: ATCTTTTGGGGTCCGTCAACT
Il6	F: TTGCCTTCTTGGGACTGATG<br>R: CCACGATTTCCCAGAGAACA
Muc2	F: GGTCCAGGGTCTGGATCACA<br>R: GCTCAGCTCACTGCCATCTG
Kcnj1	F: GGTAAGACGGTGGAAGTGGA<br>R: TTTGGGTGTCGTCTGTTTCA
Kcnma1	F: GACGTTCTGAGCGTGACTG<br>R: TGGTGGAGCAATCATTAACAGAG
Zo1	F: ACCCGAAACTGATGCTGTGGATAG<br>R: AAATGGCCGGGCAGAACTTGTGTA
Occludin	F: CTCTCAGCCAGCGTACTCTT<br>R: CTCCATAGCCACCTCCGTAG
bActin	F: AGTGTGACGTTGACATCCGT<br>R: TGCTAGGAGCCAGAGCAGTA

F: forward primer, **R**: reverse primer
